# A novel circulating miRNA panel for non-invasive ovarian cancer diagnosis and prognosis

**DOI:** 10.1038/s41416-022-01925-0

**Published:** 2022-08-05

**Authors:** Aoife Ward Gahlawat, Tania Witte, Lisa Haarhuis, Sarah Schott

**Affiliations:** 1 Department of Gynaecology and Obstetrics, University Hospital of Heidelberg, Heidelberg, Germany; 2 Present Address: National Center for Tumor Diseases (NCT), Heidelberg, Germany

**Keywords:** Tumour biomarkers, Diagnostic markers

## Abstract

**Background:**

Ovarian cancer (OC) is an aggressive disease, primarily diagnosed in late stages with only 20% of patients surviving more than 5 years after diagnosis. There is a pending need to improve current diagnostics and prognostics.

**Methods:**

In this study, we investigated total circulating cell-free microRNA (cf-miRNA) levels as well as a panel of cf-miRNAs in the plasma of OC patients (*n* = 100), patients with benign lesions (*n* = 45) and healthy controls (*n* = 99).

**Results:**

High levels of cf-miRNAs correlated with unfavourable clinical features and were an independent prognosticator of patient survival. By mining NGS data, we identified a signature panel of seven individual cf-miRNAs which could distinguish controls from benign cases with an AUC of 0.77 and controls from cancer cases with an AUC of 0.87. Importantly, in combination with the current gold-standard marker, CA-125, the panel could predict early OC with an AUC of 0.93.

**Conclusion:**

Our findings highlight the potential of cf-miRNA levels as well as individual cf-miRNAs for OC diagnosis and prognosis that warrants further clinical evaluation.

## Introduction

Ovarian cancer (OC) is a highly malignant disease with a 10-year survival rate of less than 30%, responsible for more than 200,000 deaths worldwide in 2020 [1]. Unfortunately, the vast majority of patients present with incurable advanced OC, with a dismal 5-years survival rate of <20% [2]. In contrast, women diagnosed with early-stage disease show an OS of >90%. The current standard of care for OC is tumour cytoreductive surgery followed by mainly platinum-based chemotherapeutic regimens. However, around half of the patients will develop resistance to chemotherapy or relapse [3]. Novel combinations such as the anti-VEGF antibody bevacizumab, in addition to chemotherapy, could improve the median progression-free survival by 4 months in advanced stages [4]. Therefore, there is a pending need to identify effective biomarkers for early screening, treatment response and prognosis in OC.

The serum tumour marker cancer antigen 125 (CA-125), with a sensitivity of less than 60% in early-stage OC and up to 80% in advanced stages [5] is the current gold-standard biomarker for OC diagnosis and monitoring. In addition, CA-125 has a high false positive rate in the benign setting, leading to over-diagnostic surgery [6]. While CA-125 is an attractive non-invasive marker for OC, the sensitivity and specificity must be improved to implement it as a marker for screening, particularly in early-stage disease. A combination of blood-based biomarkers might be a good solution to improve the current standard of care. For example, a panel of DNA mutations and proteins in plasma, including CA-125, was able to detect five different cancer types with a sensitivity of 69–98% and a specificity of >99% [7].

Circulating cell-free microRNAs (cf-miRNAs) have emerged as a new class of promising minimally invasive clinical biomarkers for profiling of cancer patients [8–10]. MicroRNAs are non-coding RNAs of 20–25 nucleotides long with the ability to regulate protein-coding genes by repressing translation or mRNA degradation. They are transcribed in the nucleus and exported to the cytoplasm, resulting in a mature miRNA [11]. Cf-miRNAs are remarkably stable in body fluids such as plasma, thus making them an ideal non-invasive diagnostic tool for early cancer detection [12–15]. Our group has recently shown, for the first time, that total cf-miRNA levels are an independent prognostic marker for risk stratification in breast cancer [16].

While many individual cf-miRNAs have been described as potential biomarkers in OC diagnosis and prognosis [17], a lack of standard protocols has hindered their clinical uptake. Studies have shown expression variability based on the miRNA extraction method used and among different laboratories [18]. In particular, qPCR is challenging with a lack of robust reference controls for data normalisation [19]. Therefore, novel detection methods such as next-generation sequencing (NGS) or droplet digital PCR are an attractive solution in the age of personalised medicine.

In this explorative case–control study, we evaluate whether plasma cf-miRNA levels at diagnosis can differentiate among clinical characteristics of OC and predict patients who will relapse and/or die from the disease. Using NGS data, we identify a signature of cf-miRNAs which may serve as a diagnostic panel in combination with CA-125 for early-stage OC diagnosis.

## Methods

### Ethics approval and consent to participate

Ovarian cancer (OC) patients, women who were treated for unknown pelvic mass and healthy volunteers with no known conditions were recruited at the University Hospital Heidelberg, Germany and at the National Center for Tumor Diseases (NCT), Heidelberg, Germany, between May 2015 and August 2018. All participants provided written informed consent. The study was approved by the ethics committee of the University of Heidelberg (S-046/2018, S-266/2011, S-393/201) in accordance with good clinical practice guidelines, national laws and the Declaration of Helsinki.

### Sample collection

Before surgery and chemotherapy, women filled in a questionnaire on sociodemographic information and whole blood samples were collected. The cohort is summarised in Table 1. Three EDTA tubes (Sarstedt S-Monovette K3E, 1.6 mg EDTA/ml) with 9-ml whole blood were taken. All patient information was entered into a study-specific data system (Starlims Version 10, Abbott Laboratories) after pseudonymisation.

**Table 1:** Cohort characteristics.

	Control	Benign	Cancer
*n*	99	45	100
Mean age (range)	60.1 (21–84)	58.7 (22–85)	60.3 (21–83)
Mean CA-125 (U/ml)	12.06	30.32	1035.7
Histology		*n*	*n*
Ovarian cyst		14	
Serous cystadenoma		9	
Mucinous cystadenoma		7	
Fibroma		7	
Mature teratoma		5	
Unknown		3	
Serous adenocarcinoma			80
Other			20
FIGO Stage			
I–II			30
III–IV			69
Grade			
1 and 2			17
3			74
Tumour size			
T1–T2			31
T3			53
Lymph nodes			
N0			43
N1 +			29
Metastasis			
M0			31
Local			55
M1 distant			13
Residual tumour			
R0			32
R1			10

### Plasma preparation

EDTA blood samples were centrifuged at 1300 × *g* for 20 min. The plasma fraction was further processed by high-speed centrifugation at 12,000 × *g* for 10 min. Samples were immediately stored at −80 °C.

### miRNA isolation from plasma

Circulating miRNAs were isolated from 300 µl thawed plasma using the NucleoSpin miRNA Plasma kit (Macherey-Nagel, Düren, Germany) according to the manufacturer’s protocol with the addition of 10 mg/ml glycogen. Total circulating miRNAs were quantified using the Qubit microRNA Assay Kit and the Qubit Fluorometer 3.0 (Thermo Fisher Scientific, MA, USA).

### qRT-PCR

In all, 2 µl of the miRNA eluate was synthesised to cDNA using the LNA miRNA RT kit (Qiagen, Hilden, Germany). Individual miRNAs were amplified and quantified using LNA-specific primers and the primaQUANT 2× qPCR-CYBR-Green-Blue-MasterMix (Steinbrenner, Germany) according to the manufacturer’s protocol on the qTOWER instrument (Analytical Jena, Germany). Two replicates were performed for each sample. miR-451a was measured to control for haemolysis. Commercial RNA from the breast cancer cell line MCF-7 was included on each plate as a positive control and to assess batch effects. Water was included as a negative control. Data were either normalised to the global mean or analysed as raw Ct values.

### CA-125 measurement

CA-125 was measured in the plasma of healthy control subjects using the Human CA-125/MUC16 Quantikine ELISA Kit (R&D Systems, USA) according to the manufacturer’s instructions. The final concentration was calculated in U/ml according to the given standards.

### Statistical analysis

For prognostic analysis of total cf-miRNA levels, Kaplan–Meier survival plots were computed with GraphPad prism version 8.0. All other analyses were performed using R version 4.1.2. In order to compute univariate hazard ratios, a Cox proportional hazards regression model [20] was fitted for each parameter separately, with the respective overall or progression-free survival time as a dependent variable and the respective marker as a single covariate. Multivariate hazard ratios are stemming from a multivariate Cox proportional hazards regression model, where all parameters simultaneously were used as covariates when fitting the Cox models for overall or progression-free survival. For all Cox models, *P* values for the null hypothesis that the hazard ratio equals to 1 were derived by means of a standard Wald test.

For diagnostic analysis, CA-125 values were used on a log scale as they showed extreme outliers. Missing CA-125 values were replaced by the mean value of the corresponding group (i.e., control, benign or case).To identify the predictive value of the miRNA signature, multinomial logistic regression was carried out for the control group compared to the benign and case groups. For the final model, makers which had no significant effect in any comparison were removed. In the second step, multinomial logistic regression was repeated for the control, compared to the early and late-stage cancer groups. To determine sensitivity and specificity, a bootstrapping method with 500 repetitions was carried out whereby the data was split into 80% training and 20% testing data. The ROC curves presented are based on the complete dataset. The REMARK (Reporting Recommendations for Tumour Marker Prognostic Studies) guidelines were implemented to report results [21].

## Results

### Cohort characteristics

The cohort (*n* = 244) consisted of 100 OC cases, primarily of serous adenocarcinoma (*n* = 80), 99 age-matched healthy controls and 45 patients with various benign ovarian lesions, as summarised in Table 1. The majority of patients presented with advanced FIGO Stage III–IV (*n* = 69), high grade 3 (*n* = 74) and larger tumours (T3 *n* = 53) (Table 1). Follow-up information was collected up to 3.8 years post diagnosis with an average of 1.8 years. In the follow-up period, 31 patients were diagnosed with a progressive disease and 15 died from their disease.

### Higher cf-miRNA levels correlate with adverse clinical features

While many individual cf-miRNAs have been studied in OC [22], few studies until now have addressed the impact of global levels of cf-miRNAs. We have recently shown that total circulating miRNA levels can be used as an independent prognostic marker in breast cancer and correlate with adverse tumour characteristics [16]. To assess whether increased levels of global cf-miRNAs are associated with clinical features of OC, patients were dichotomised according to the median into either “low” of “high” cf-miRNA groups. The clinicopathological characteristics according to cf-miRNA levels are presented in Table 2. CA-125 levels were dichotomised at 35 U/ml which is considered an appropriate cut-off for OC [23]. Higher levels of cf-miRNA at cancer diagnosis were significantly associated with higher CA-125 (*P* = 0.02) and the presence of distant metastasis (*P* = 0.04). There was also a tendency for higher cf-miRNA levels to associate with advanced FIGO stage (*P* = 0.08), larger tumour size (*P* = 0.06) and the presence of residual tumour after surgery (*P* = 0.07), although it did not reach significance. Interestingly, higher levels of cf-miRNA were most significantly associated with patients' death (*P* = 0.0001), but not with relapse (Table 2).

**Table 2:** Clinicopathological characteristics of patients according to cf-miRNA levels.

Characteristics		Low cf-miRNA	High cf-miRNA	
		*n* (%)	*n* (%)	*P*
Age	<50	15 (15)	9 (9)	0.240
	>50	35 (35)	41 (41)	
CA-125	<35 U/ml	11 (12)	3 (3)	**0.020**
	>35 U/ml	34 (37)	43 (47)	
FIGO Stage	I– II	19 (20)	10 (10)	0.080
	III–IV	31 (32)	37 (38)	
Grade	1–2	9 (10)	8 (9)	ns
	3	39 (43)	35 (38)	
Tumour size	T1–T2	21 (25)	10 (12)	0.060
	T3	24 (29)	29 (35)	
Lymph nodes	N0	24 (33)	19 (26)	ns
	N1+	14 (19)	15 (21)	
Metastasis	M0	49 (49)	40 (40)	**0.040**
	M1	2 (2)	8 (8)	
Residual tumour	R0	18 (43)	14 (33)	0.070
	R1	2 (5)	8 (19)	
Relapse	Yes	14 (14)	17 (17)	ns
	No	37 (37)	31 (31)	
Death	Yes	1 (1)	14 (14)	**0.0001**
	No	50 (51)	34 (34)	

*P* values were obtained using Fisher’s exact test for categorical factors.

Significant *P* values are shown in bold.

### Total cf-miRNA is an independent prognostic marker for survival in ovarian cancer

**Fig. 1:**
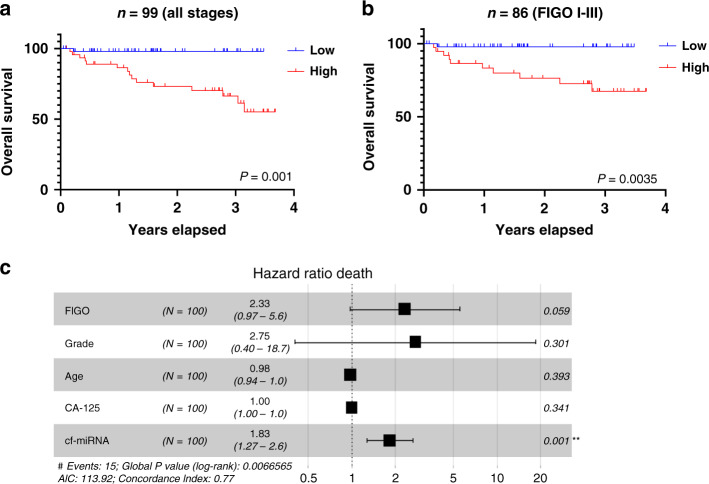
Circulating miRNAs are an independent prognostic marker in OC. Patients were dichotomised based on the median cf-miRNA levels and associations with overall survival are depicted after Kaplan–Meier survival analysis (**a**). The same analysis was performed, excluding patients with FIGO Stage 4 disease (**b**). *P* values shown represent the statistical significance between the two curves. Multivariate Cox regression analysis for overall survival is presented as a forest plot (**c**).

Because higher levels of circulating miRNAs correlated with adverse clinical features of OC, we hypothesised that they could also have a prognostic impact. To this end, we carried out Kaplan–Meier analysis for overall survival (OS) and progression-free survival (PFS) in the cancer cohort. Follow-up information was available for up to 3 years and 8 months post diagnosis (median time 1 year and 8 months). Higher levels of baseline cf-miRNA were significantly associated with OS (*P* = 0.001, Fig. 1a) but not with PFS (Supplementary Fig. 1A). Since patients with advanced OC have the shortest survival time [2], we repeated the analysis on just the FIGO I-III patients and could recapitulate the results (*P* = 0.0035, Fig. 1b). To validate these findings, we carried out univariate and multivariate Cox regression analysis. Patient age, tumour grade, FIGO stage, residual tumour and CA-125 were included as outcome variables. Since information on the residual tumour was only available for a subset of patients (*n* = 42), we did not include this factor in the multivariate analysis. In line with the Kaplan–Meier analysis, cf-miRNA levels were highly significant for OS in both univariate (HR 1.89, *P* = 0.00007) and multivariate (HR 1.83, *P* = 0.001) analysis (Fig. 1c and Supplementary Table 1), confirming that total cf-miRNA is an in the independent prognostic marker for survival in OC. Cf-miRNA was not associated with PFS (Supplementary Fig. 1b and Supplementary Table 1).

### A panel of seven cf-miRNAs for OC diagnosis

**Fig. 2:**
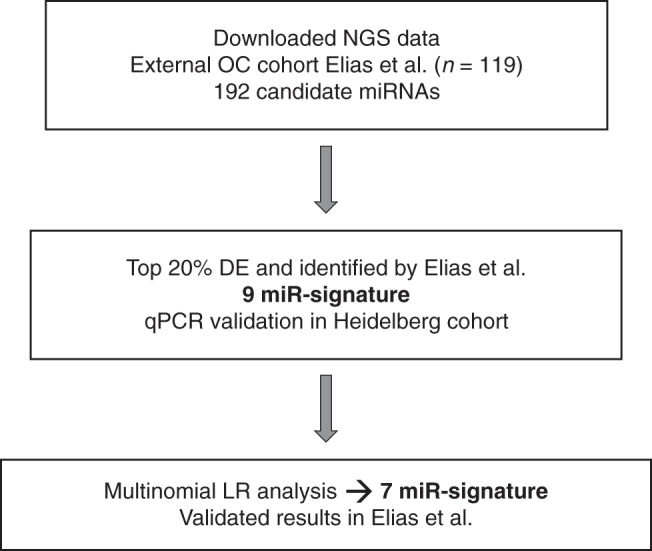
Seven miR-signature panel selection. A flow-through of how the signature panel of cf-miRNAs for OC diagnosis was selected. A list of 192 differentially expressed cf-miRNAs in OC were retrieved leading to a 9-miR signature for qPCR validation which was reduced to final panel of 7 after multinomial logistic regression analysis.

Since total cf-miRNA levels had a significant prognostic impact on OC survival, we hypothesised that they may also have diagnostic potential. To this end, we compared cf-miRNA levels in the three groups: healthy controls, benign and cancer cases. Because cf-miRNA levels were not normally distributed (Supplementary Fig. 1C), we performed a log transformation of the data and found no significant difference between the three groups (Supplementary Fig. 1D), indicating that cf-miRNA levels are not an appropriate diagnostic marker of OC. Next, we carried out an extensive literature search to identify candidate cf-miRNAs which might have diagnostic potential in OC. We decided to focus our efforts on one study in particular due to the large cohort size and the implementation of miRNA sequencing technology, from which the data were available for download (Fig. 2) [24]. We believe that NGS is more robust for plasma profiling since circulating miRNAs are generally lowly expressed compared to tissues and with NGS one has the ability to sequence the whole genome while microarrays have a predefined set of transcripts. In the aforementioned study, 192 cf-miRNAs were eligible for analysis after filtering out lowly expressed cf-miRNAs. Next, we selected the top 20% differentially expressed miRNAs between cases and controls and cross-checked which of those miRNAs had also been selected for diagnosis in the original study [24], which resulted in a 9-miR signature (Fig. 2).

**Fig. 3:**
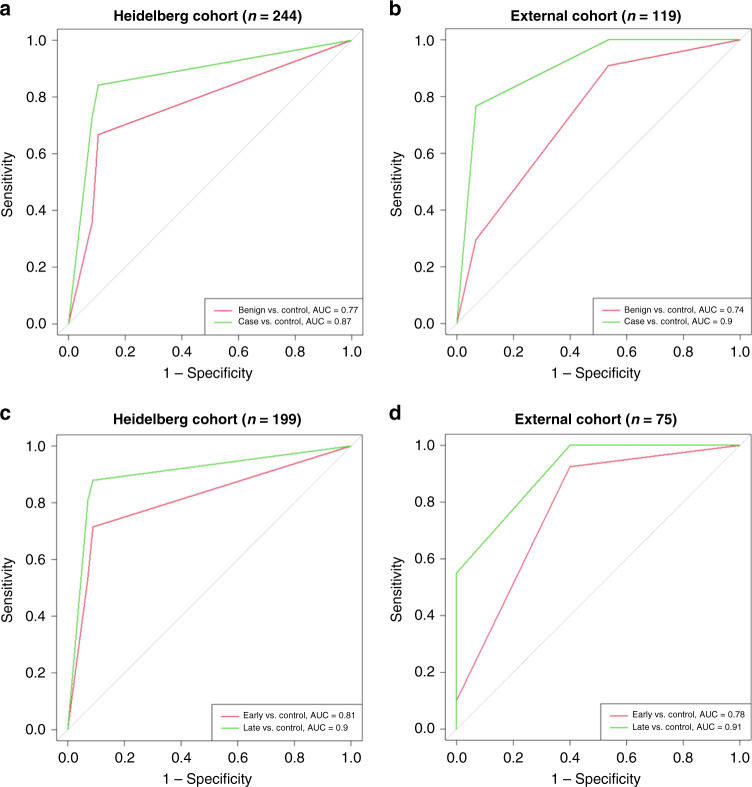
A 7-miR panel to detect OC. AUC curves depict the diagnostic ability of a signature panel of seven cf-miRNAs to detect benign (red line) and OC cases (green line) (**a**, **b**) and to distinguish early (red line) and late (green line) cases from controls (**c**, **d**) in two independent cohorts.

To investigate the predictive value of this miRNA signature in our cohort, we measured the nine cf-miRNAs by qRT-PCR and performed multinomial logistic regression analysis. The expression of the nine cf-miRNAs mostly positively correlated with each other (Supplementary Table 2). Because there is no standard method for normalisation of cf-miRNA data [19], we performed the analysis using either the raw Ct values or applied a method for global normalisation. As both methods produced similar results (data not shown), we decided to consider the raw Ct values. We first tested the impact of each individual miRNA when comparing controls to either benign or cancer cases. This resulted in a final seven miR signature of miR-92a, -200c, -320b, -320c, -335, -375, -486 for further analysis. The final 7-miR signature could predict benign cases from controls with an area under the curve (AUC) of 0.77 and OC cases with an AUC of 0.87 (Fig. 3a). Next, we wanted to assess the diagnostic potential of cf-miRNAs specifically in early-stage OC. Interestingly, the cf-miRNA signature could detect early cancer cases with an AUC of 0.81 which increased to 0.9 for late-stage disease (Fig. 3c and Supplementary Table 3). The results could be reproduced in the external cohort (Fig. 3b, d, Supplementary Table 3).

### A panel of cf-miRNAs combined with CA-125 for early OC detection

**Fig. 4:**
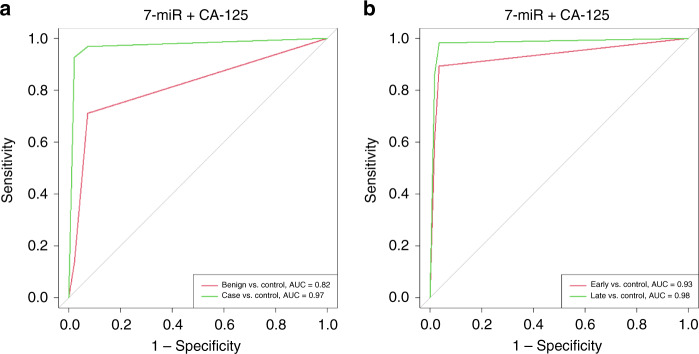
cf-miRNA in combination with CA-125 for OC diagnosis. AUC curves depict the diagnostic ability of cf-miRNAs in combination with CA-125 for benign (red line) and OC (green line) diagnosis (**a**) and early (red line) and late-stage (green line) OC diagnosis (**b**).

Finally, we asked if the diagnostic ability of cf-miRNAs could be improved with the addition of the current gold-standard biomarker, CA-125. In combination with CA-125, the 7-miR signature could distinguish cases from controls with an AUC of 0.97 which was retained with an AUC of 0.93 for early-stage OC (Fig. 4 and Supplementary Table 3). This result highlights the potential of cf-miRNAs to improve the current diagnostic methods for early-stage OC which could have a true impact on disease progression.

## Discussion

In this study, we have validated the potential of cf-miRNAs as diagnostic and prognostic markers in OC. Our results show, for the first time, that total cf-miRNA levels are associated with adverse clinical features in OC and are an independent prognostic marker for survival. In addition, a panel of seven cf-miRNAs could distinguish cancer cases from healthy controls with an AUC of 0.87 which was further improved to 0.97 with the addition of CA-125, and retained with an AUC of 0.93 for early-stage OC detection. Our findings highlight the potential of cf-miRNAs in OC diagnosis and prognosis, where there is a need for improved biomarkers.

Much of the current liquid biopsy research focuses on circulating tumour cells (CTCs) or circulating tumour DNA (ctDNA) which are thought to be released directly from the tumour [25] and therefore are discussed as tumour-specific biomarkers. However, a recent review on in OC outlined the limitations and divergent findings in several studies [26]. Our work demonstrates that cf-miRNAs are also ideal candidates as liquid biopsy-based markers that could be easily applied in the clinical setting. Recently, our group has shown that levels of cf-miRNAs are an independent prognostic marker in breast cancer and correlate with unfavourable tumour features [16]. Here, we have shown very similar results in an OC cohort. These findings indicate, for the first time, that levels of cf-miRNAs could be prognostic for additional cancer types, which warrants further investigation.

For total cf-miRNA levels, we have used the simple Qubit instrument which could easily be adapted in the clinic, it is cost-effective and requires less than 1 ml of blood. In comparison to the other clinical features of OC tested, only cf-miRNA levels were significant for survival in multivariate analysis, highlighting its prognostic impact. In addition, cf-miRNAs are very stable in blood which makes them an ideal liquid biopsy marker [15]. In contrast to our findings in breast cancer [16], cf-miRNA levels did not associate with OC prognosis. In the relatively short follow-up time (average 1.8 years) more than 30% of patients relapsed. We hypothesise that since our cohort was skewed towards more advanced OC, cf-miRNA levels at diagnosis were not informative for progression. Nevertheless, we would be interested to analyse cf-miRNA levels in longitudinal samples to assess whether they are informative in detecting residual disease and/or therapy response.

Since global cf-miRNA levels did not differ between healthy controls, benign and cancer cases, we investigated a panel of individual plasma miRNAs, derived from a previously published cohort [24]. This resulted in a signature of seven individual miRNAs for OC diagnosis: miR-92a, -200c, -320b, -320c, -335, -375, -486. Interestingly, miR-200c was the top upregulated cf-miRNA in the NGS dataset and the authors could also show a significant reduction after surgery, indicating that this miRNA is actively produced by OC tumours [24]. In line with these results, miR-200c and other members of the miR-200 family were found to have significantly higher serum concentrations in OC when compared to healthy women with an AUC of 0.828 [27]. Similarly, Kan et al. found members of the miR-200 family to be upregulated in OC serum (*n* = 28) compared to healthy controls (*n* = 28) with an AUC of 0.784 [28]. One study also found members of the miR-200 family to be associated with OS and PFS, further suggesting their functionality beyond being a biomarker in OC [29]. However, most of these studies had a relatively low number of patients.

In one of the largest studies to date, over 4000 serum samples from OC and healthy controls were screened by microarrays to develop a model of 10 diagnostic cf-miRNAs (sensitivity 0.99 and specificity 1.0), which was maintained for early-stage OC. Interestingly, miR-320a was one of the best predictors with an AUC of 0.96 [30]. Our signature panel also contained two members of the mir-320 family. Recent work from Cirillo and colleagues have investigated the expression of miR-141 (from the miR-200 family) and miR-320b in combination with two protein markers, CA-215 and HE4 for OC diagnosis [31]. Similar to our findings, the addition of protein biomarkers markedly improved the model performance (sensitivity = 88.9%, specificity = 100%, AUC = 1.000). In line with this, Oliveira et al. could distinguish benign from malignant tumours with an overall diagnostic accuracy of AUC 0.96 using just two candidate miRNAs in combination with CA-125 [32]. In our current work, adding CA-125 remarkably improved the AUC for all groups tested. Taken together, this confirms that cf-miRNAs have great potential to be used alongside the current standard diagnostics for OC. By implementing a simple blood test, women at high risk, or with suspected OC could be identified at an early stage, when the survival odds are highest.

A major limitation of our study is that we did not perform our own miRNA profiling, but relied on data from another study. With this approach, we cannot be sure that the selected miRNAs are the most representative of our cohort. On the other hand, the external data served as a validation cohort in our study. In addition, the external dataset was based on NGS which limits any potential technical bias from the qPCR analysis [18]. While we could validate the 7-miR signature, we did not have the additional layers of CA-125 for early-stage diagnosis or cf-miRNA levels for prognostic analysis. Therefore, we would like to confirm our findings in future work on an independent cohort. Another limitation is that our panel signature was optimised to distinguish cases from healthy controls, rather than from benign cases. It would be of interest to carry out a more robust screening method in future studies to identify markers of benign lesions, which are mainly diagnosed with invasive surgery. The samples for our study were collected and processed together at one centre, which reduces variability, yet limits our findings. In addition, we had a relatively low follow-up time which may have hindered the prognostic studies. Our cancer cohort was also imbalanced, with an overrepresentation of late-stage serous carcinomas. However, we had quite a large sample size in comparison to similar studies and an age-matched control cohort for accurate analyses.

In conclusion, we believe that our findings pave the way to the clinical evaluation of a cf-miRNA-based liquid biopsy for OC diagnosis and prognosis in addition to the current standard of care. The results should be evaluated in prospective larger-scale clinical studies to determine the clinical utility for women, particularly in high-risk settings.

## Supplementary information

Supplementary file

REMARK

## Data Availability

Not applicable.
