# Supplementary material for: A novel circulating miRNA panel for non-invasive ovarian cancer diagnosis and prognosis
Source: Br J Cancer. 2022 Aug 5;127(8):1550–6. doi: 10.1038/s41416-022-01925-0 (PMC9553930; doi:10.1038/s41416-022-01925-0)
Supplement: Supplementary file 1 — Supplementary file [file 41416_2022_1925_MOESM1_ESM.pptx]

## Slide 1
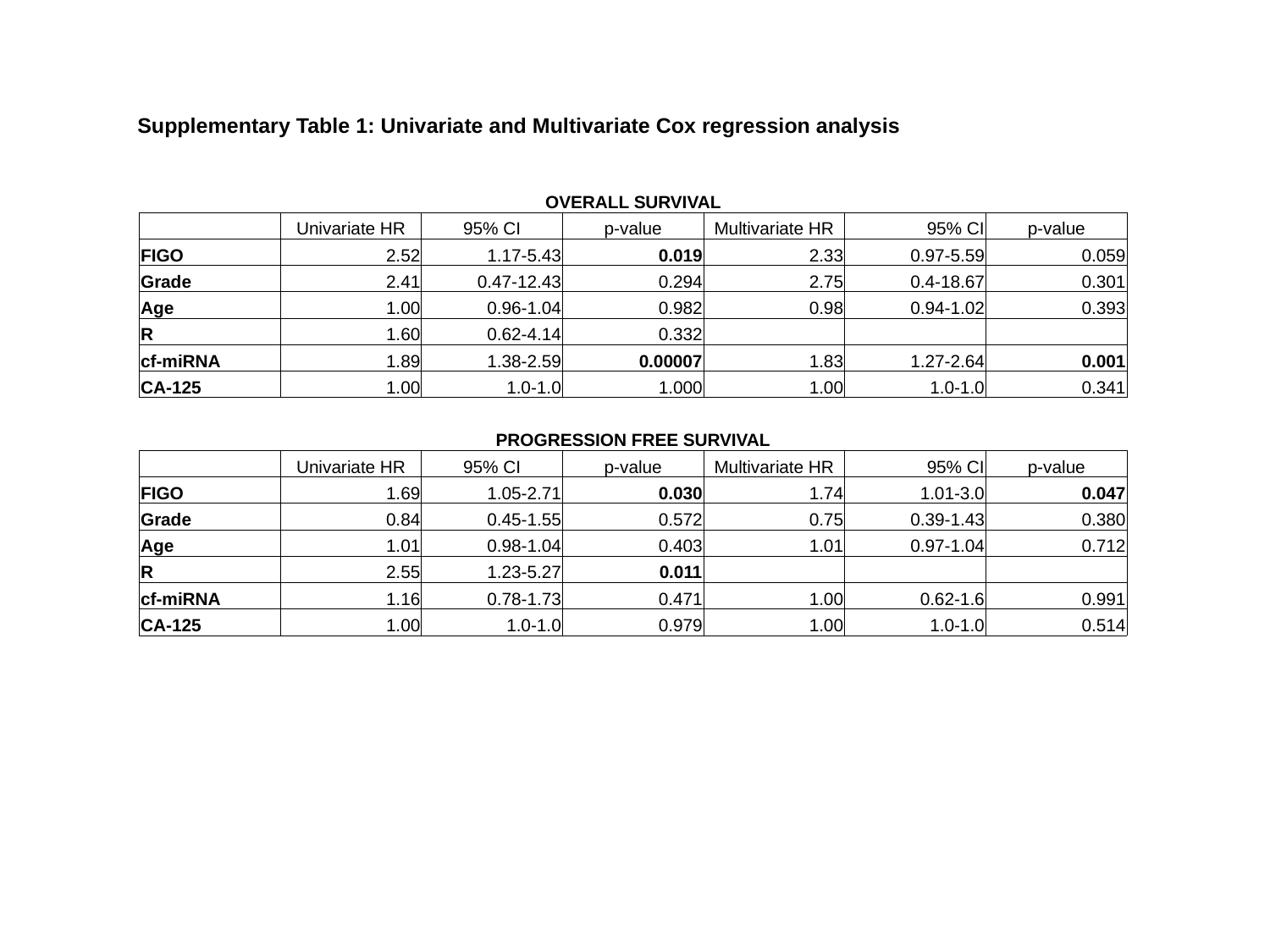

Supplementary Table 1: Univariate and Multivariate Cox regression analysis
| OVERALL SURVIVAL | | | | | | |
| --- | --- | --- | --- | --- | --- | --- |
| | Univariate HR | 95% CI | p-value | Multivariate HR | 95% CI | p-value |
| FIGO | 2.52 | 1.17-5.43 | 0.019 | 2.33 | 0.97-5.59 | 0.059 |
| Grade | 2.41 | 0.47-12.43 | 0.294 | 2.75 | 0.4-18.67 | 0.301 |
| Age | 1.00 | 0.96-1.04 | 0.982 | 0.98 | 0.94-1.02 | 0.393 |
| R | 1.60 | 0.62-4.14 | 0.332 | | | |
| cf-miRNA | 1.89 | 1.38-2.59 | 0.00007 | 1.83 | 1.27-2.64 | 0.001 |
| CA-125 | 1.00 | 1.0-1.0 | 1.000 | 1.00 | 1.0-1.0 | 0.341 |
| | | | | | | |
| PROGRESSION FREE SURVIVAL | | | | | | |
| | Univariate HR | 95% CI | p-value | Multivariate HR | 95% CI | p-value |
| FIGO | 1.69 | 1.05-2.71 | 0.030 | 1.74 | 1.01-3.0 | 0.047 |
| Grade | 0.84 | 0.45-1.55 | 0.572 | 0.75 | 0.39-1.43 | 0.380 |
| Age | 1.01 | 0.98-1.04 | 0.403 | 1.01 | 0.97-1.04 | 0.712 |
| R | 2.55 | 1.23-5.27 | 0.011 | | | |
| cf-miRNA | 1.16 | 0.78-1.73 | 0.471 | 1.00 | 0.62-1.6 | 0.991 |
| CA-125 | 1.00 | 1.0-1.0 | 0.979 | 1.00 | 1.0-1.0 | 0.514 |

## Slide 2
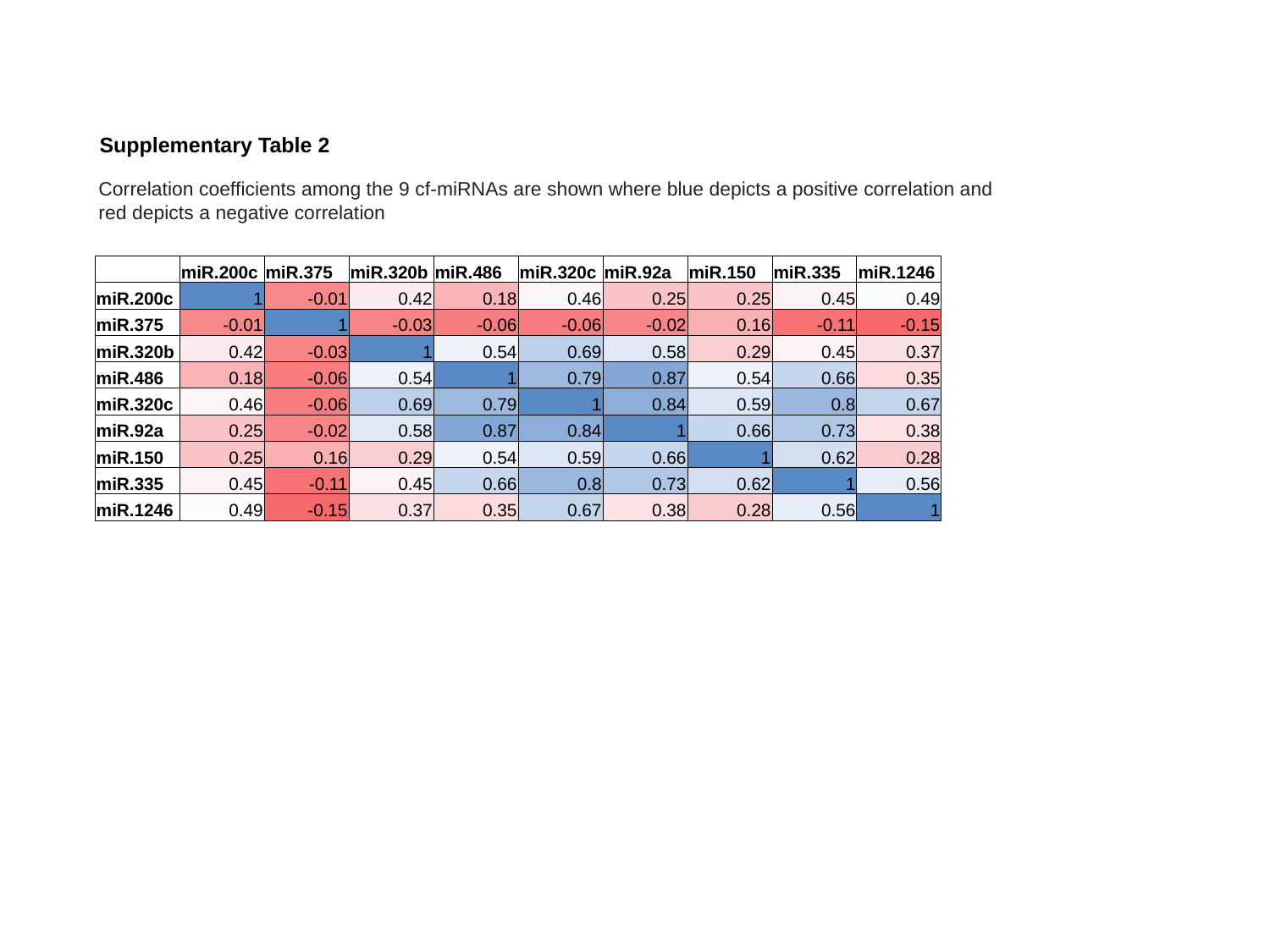

Supplementary Table 2
Correlation coefficients among the 9 cf-miRNAs are shown where blue depicts a positive correlation and red depicts a negative correlation
| | miR.200c | miR.375 | miR.320b | miR.486 | miR.320c | miR.92a | miR.150 | miR.335 | miR.1246 |
| --- | --- | --- | --- | --- | --- | --- | --- | --- | --- |
| miR.200c | 1 | -0.01 | 0.42 | 0.18 | 0.46 | 0.25 | 0.25 | 0.45 | 0.49 |
| miR.375 | -0.01 | 1 | -0.03 | -0.06 | -0.06 | -0.02 | 0.16 | -0.11 | -0.15 |
| miR.320b | 0.42 | -0.03 | 1 | 0.54 | 0.69 | 0.58 | 0.29 | 0.45 | 0.37 |
| miR.486 | 0.18 | -0.06 | 0.54 | 1 | 0.79 | 0.87 | 0.54 | 0.66 | 0.35 |
| miR.320c | 0.46 | -0.06 | 0.69 | 0.79 | 1 | 0.84 | 0.59 | 0.8 | 0.67 |
| miR.92a | 0.25 | -0.02 | 0.58 | 0.87 | 0.84 | 1 | 0.66 | 0.73 | 0.38 |
| miR.150 | 0.25 | 0.16 | 0.29 | 0.54 | 0.59 | 0.66 | 1 | 0.62 | 0.28 |
| miR.335 | 0.45 | -0.11 | 0.45 | 0.66 | 0.8 | 0.73 | 0.62 | 1 | 0.56 |
| miR.1246 | 0.49 | -0.15 | 0.37 | 0.35 | 0.67 | 0.38 | 0.28 | 0.56 | 1 |

## Slide 3
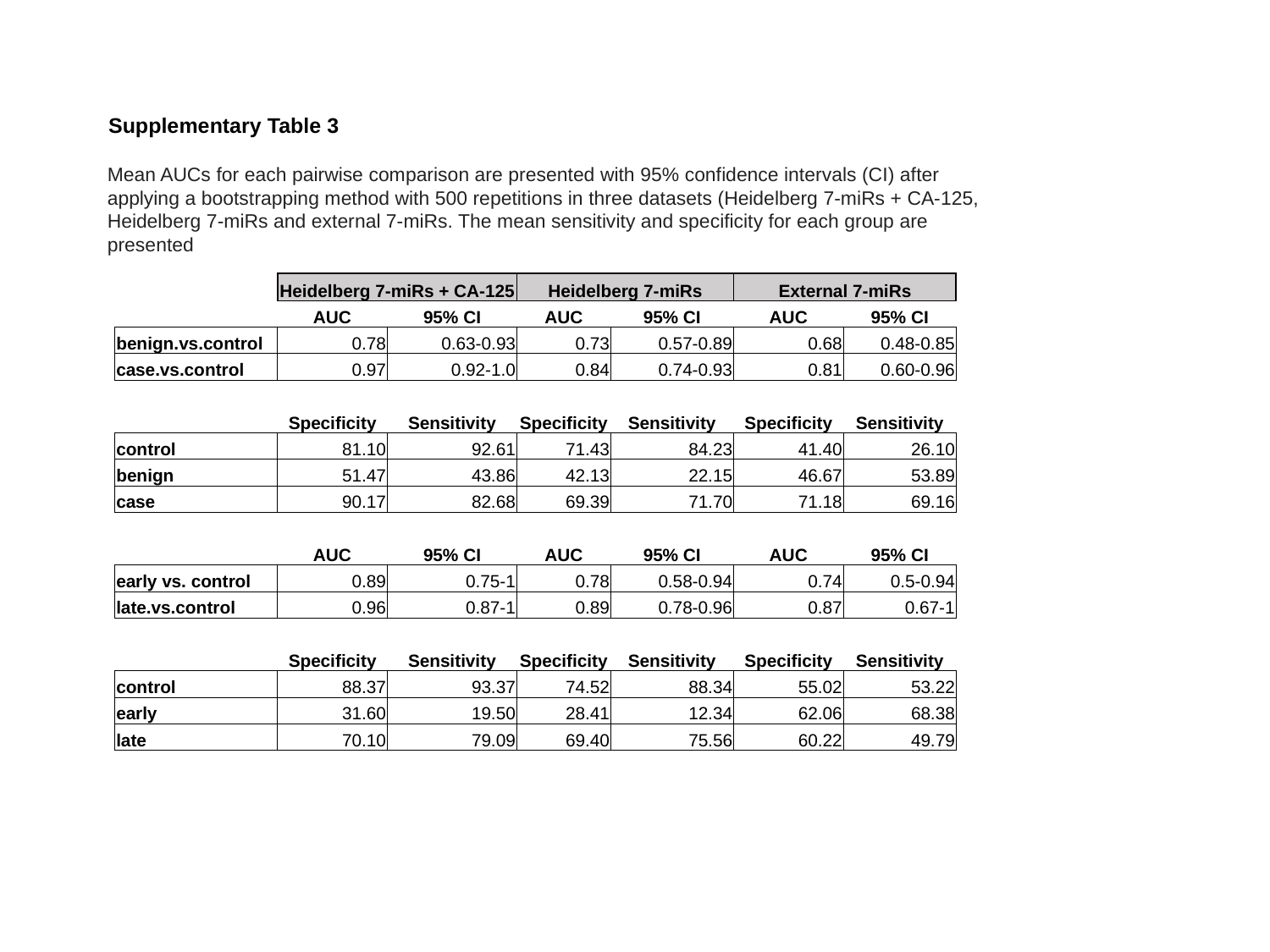

Supplementary Table 3
Mean AUCs for each pairwise comparison are presented with 95% confidence intervals (CI) after applying a bootstrapping method with 500 repetitions in three datasets (Heidelberg 7-miRs + CA-125, Heidelberg 7-miRs and external 7-miRs. The mean sensitivity and specificity for each group are presented
| | | | | | | |
| --- | --- | --- | --- | --- | --- | --- |
| | Heidelberg 7-miRs + CA-125 | | Heidelberg 7-miRs | | External 7-miRs | |
| | AUC | 95% CI | AUC | 95% CI | AUC | 95% CI |
| benign.vs.control | 0.78 | 0.63-0.93 | 0.73 | 0.57-0.89 | 0.68 | 0.48-0.85 |
| case.vs.control | 0.97 | 0.92-1.0 | 0.84 | 0.74-0.93 | 0.81 | 0.60-0.96 |
| | | | | | | |
| | Specificity | Sensitivity | Specificity | Sensitivity | Specificity | Sensitivity |
| control | 81.10 | 92.61 | 71.43 | 84.23 | 41.40 | 26.10 |
| benign | 51.47 | 43.86 | 42.13 | 22.15 | 46.67 | 53.89 |
| case | 90.17 | 82.68 | 69.39 | 71.70 | 71.18 | 69.16 |
| | | | | | | |
| | AUC | 95% CI | AUC | 95% CI | AUC | 95% CI |
| early vs. control | 0.89 | 0.75-1 | 0.78 | 0.58-0.94 | 0.74 | 0.5-0.94 |
| late.vs.control | 0.96 | 0.87-1 | 0.89 | 0.78-0.96 | 0.87 | 0.67-1 |
| | | | | | | |
| | Specificity | Sensitivity | Specificity | Sensitivity | Specificity | Sensitivity |
| control | 88.37 | 93.37 | 74.52 | 88.34 | 55.02 | 53.22 |
| early | 31.60 | 19.50 | 28.41 | 12.34 | 62.06 | 68.38 |
| late | 70.10 | 79.09 | 69.40 | 75.56 | 60.22 | 49.79 |

## Slide 4
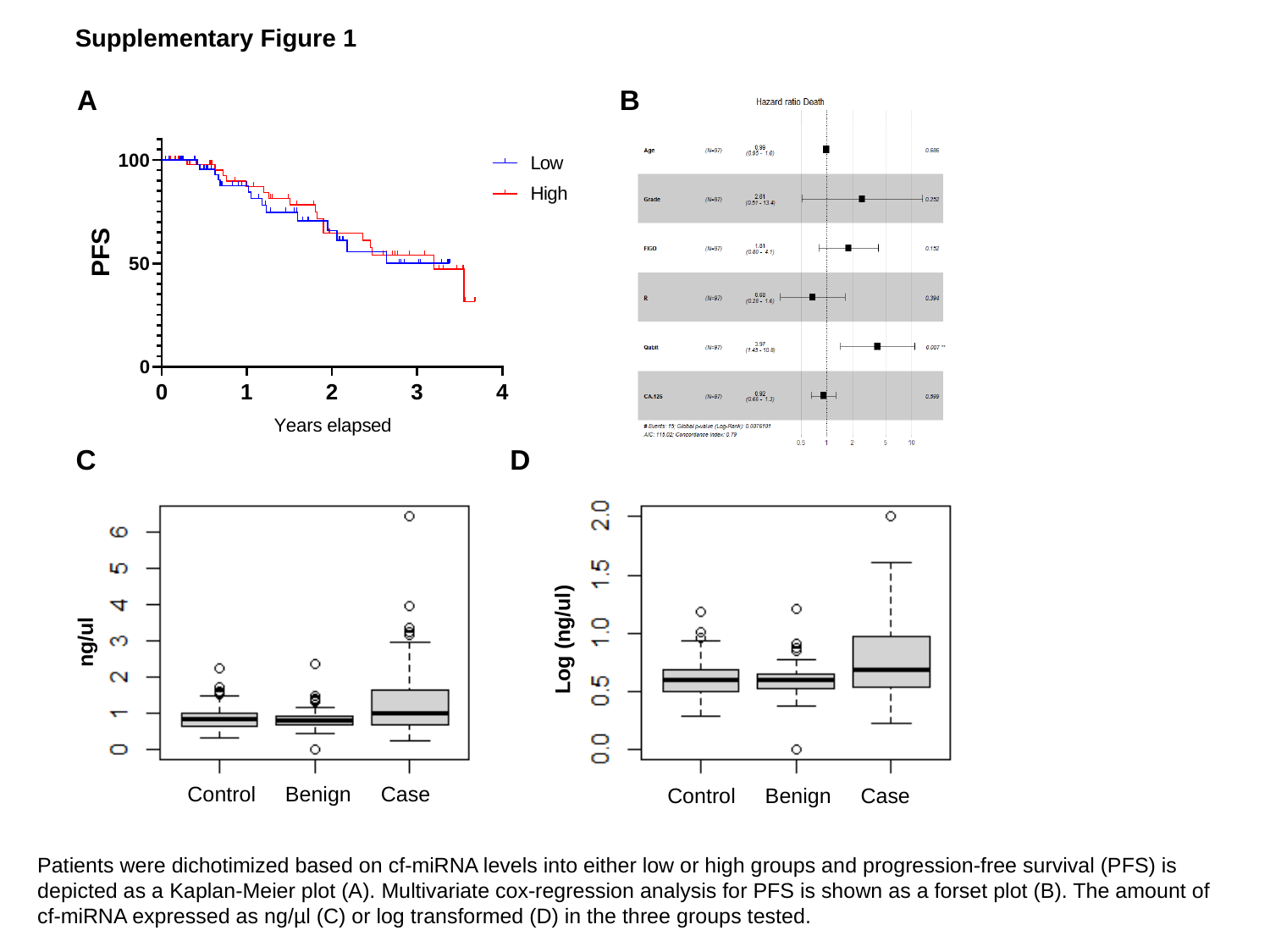

Supplementary Figure 1
A B
C D
Log (ng/ul)
ng/ul
Control Benign Case
Control Benign Case
Patients were dichotimized based on cf-miRNA levels into either low or high groups and progression-free survival (PFS) is depicted as a Kaplan-Meier plot (A). Multivariate cox-regression analysis for PFS is shown as a forset plot (B). The amount of cf-miRNA expressed as ng/µl (C) or log transformed (D) in the three groups tested.
